# Recurrence of Chronic Myeloid Leukemia during Pregnancy Subsequently Achieving Complete Medical Remission

**DOI:** 10.1155/2017/5651064

**Published:** 2017-08-03

**Authors:** Sasha Mikhael, Ashlee Pascoe, Joseph Prezzato

**Affiliations:** Department of Obstetrics and Gynecology, Providence Hospital, Michigan State University College of Human Medicine, East Lansing, MI, USA

## Abstract

The treatment of chronic myeloid leukemia (CML) with tyrosine kinase inhibitors (TKIs) in reproductive-aged women poses major dilemmas concerning its associated teratogenicity as observed in many animal studies. Much controversy exists regarding continuation versus discontinuation of its use in pregnancy with some studies suggesting safety of TKIs before and during pregnancy and others reporting toxicity and adverse outcomes. TKIs have become a well-established treatment option for CML, significantly improving prognosis, and yet have been reported to be fetotoxic. We present a case of a 25-year-old woman who achieved successful pregnancy and delivery after withholding treatment, meanwhile relapsing, eventually achieving complete molecular remission after reinitiation of high dose dasatinib.

## 1. Introduction

Chronic myeloid leukemia (CML) in pregnancy has posed great disparity due to the lack of standard guidelines for its management and concerns regarding effects of teratogenicity. The bcr-abl tyrosine kinase gene mutation, acquired from reciprocal translocation of chromosomes 9 and 22, known as the Philadelphia chromosome, has long been established as the mechanism causing the development of CML. This has led to the development of tyrosine kinase inhibitors (TKIs). TKIs have revolutionized treatment of CML, improving prognosis and thereby its survival to as much as 85% [1]. Unfortunately, almost 25% of patients diagnosed with CML are of reproductive age [2] creating a dilemma regarding treatment approach due to the associated teratogenicity observed in many animal studies [3]. There continues to be controversy in the literature regarding how to counsel patients, specifically on when to withhold medications and for how long it is safe to do so. We describe a case of a 25-year-old woman with a 4-year long history of CML who discontinued treatment for the purpose of conception and successfully delivered a healthy baby, meanwhile relapsing.

## 2. Case Report

A 25-year-old gravid 2 para 1 with a known history of CML presented to the office for preconception counseling in December 2015. The patient was diagnosed with CML in January 2013 after presenting to the emergency department with symptoms of severe generalized pain and intractable vomiting. Upon work-up, her white blood cell count was found to be significantly elevated (value of 51.7 × 10^9^/L), prompting repeat of lab values. With further investigation via bone marrow biopsy, she was confirmed to have CML and was immediately referred to an oncologist. Shortly thereafter, treatment with Sprycel (dasatinib) was initiated, a second-generation TKI, allowing the patient to successfully achieve cytogenetic remission 10 months after initiation of therapy in January of 2013. She reported adverse effects from the medication including joint pain, morning emesis, and hair loss that she described as tolerable. The patient finally achieved and remained in complete molecular remission with continuation of dasatinib. In December 2015, she sought preconception counseling at our office where she was referred back to her oncologist who was agreeable to discontinuation of dasatinib in order to allow for conception to minimize risk of teratogenicity, particularly during organogenesis. The patient immediately had her Mirena intrauterine device removed and subsequently had a positive pregnancy test nearing the end of January 2016, only one month after discontinuation of dasatinib. She was seen back in our office on February 10, 2016, for her first prenatal visit. Prenatal labs were drawn and she was found to have an intrauterine pregnancy of 6 weeks and 5 days of gestation with a fetal heart rate of 125 bpm. All prenatal labs were within normal limits with a hemoglobin level of 13 g/dl, white blood cell count of 7.3 10^9^/L, integrated genetic screening, and a targeted anatomy scan at 19 weeks of gestation showing no detectable abnormalities. The patient continued to follow closely with her oncologist every 3 months. At approximately 25 weeks of gestation, repeat quantitative real time polymerase chain reaction (qRT-PCR) of peripheral blood smear demonstrated elevation of bcr-abl mutation above baseline from remission. The patient was also referred for a maternal fetal medicine consultation who recommended close follow-up every 3-4 weeks for serial ultrasonography to follow growth in addition to antenatal fetal surveillance between 32 and 34 weeks of gestation. Both maternal fetal medicine specialists and her oncologist agreed to induce labor at 36-37 weeks of gestation. Serial ultrasonography showed normal growth and amniotic fluid index with persistent 8/8 biophysical profiles and category 1 tracings on nonstress tests indicating reassuring fetal status. At 37 weeks of gestation, we proceeded with an induction of labor with successful delivery of a healthy baby boy weighing 6 pounds and 12 ounces via uncomplicated vaginal delivery with APGARS of 8 and 9, at 1 and 5 minutes, respectively. She continued to breastfeed for a duration of 2 weeks until her follow-up appointment, where she was restarted on dasatinib at a high dose of 100 mg.

The patient continued to do well with follow-up qRT-PCR testing in March 28, 2017, indicating complete molecular response (0.000%). To prevent future pregnancies, she had a Mirena IUD replaced and her partner underwent a vasectomy as she will require lifelong therapy in hopes of maintaining complete molecular remission.

## 3. Discussion

There are many conventional therapeutic options for the management of CML including hydroxyurea, busulfan, interferon based regimens, leukapheresis, and stem cell transplantation (or bone marrow transplant) [3], with bone marrow transplantation being curative. Cytotoxic drugs such as hydroxyurea (HU) and interferon have proven to be safe in pregnancy, HU having the lowest fetotoxic effects, but they do not possess the same efficacy as TKIs to induce hematologic and cytogenetic remission [3]. Tyrosine kinase inhibitors have been shown to be efficacious as seen with our patient who rapidly achieved molecular remission after initiation of dasatinib. Though specific guidelines are not yet available, many studies and case reports have shown that it is possible to advise patients to discontinue treatment for purposes of conception and pregnancy in the event that a complete medical response has been achieved if there is adequate counseling and patients are followed closely, according to the French-STIM study [4]. A minimum duration of 2 years of remission appears to be the safest to proceed with discontinuation of TKI, as was advised to this patient. In fact, it appears that patients who have achieved complete molecular remission as defined by nondetectable levels of bcr-abl mRNA for a duration of two years may discontinue therapy altogether, suggesting patients may be cured of CML without requiring bone marrow transplantation [5]. Though there is no consensus on the management of CML in pregnancy, much of the cited literature advises against use of TKI due to risk of embryo-fetal toxicity resulting in malformations, spontaneous abortion, and fetal growth restriction [1–3, 6]. Some of the reported malformations in animal studies included skeletal and soft tissue abnormalities [6] where specifically dasatinib was observed to be in high concentrations in maternal tissue and breast milk. Few studies have recommended restarting imatinib during the second trimester or using drugs that are less fetotoxic such as interferon or hydroxyurea but this has not been widely studied [3]. With the limited information currently present in the medical literature, it appears that there is general consensus to encourage preconception counseling with planned conception followed by serial ultrasonography and molecular testing. Continued monitoring of bcr-abl by qRT-PCR analysis has proven to be helpful. Patients should be heavily informed on the risk of relapse when interrupting TKI during pregnancy. It appears that it is safe to breastfeed for a short duration with the goal to primarily provide colostrum to the neonate due to benefits of providing nutrients and passive immunity, transmission of cytokines and growth factors, and decreasing constipation by clearing excess bilirubin. Perhaps further studies could provide a more defined therapeutic approach for reproductive-aged women looking to conceive. In addition, continued investigation could allow for a deeper understanding of the safety profile of TKIs in pregnancy to allow for its continuation in order to minimize risk of recurrence, meanwhile providing safety in pregnancy and delivery.

## References

[1] Pavlovsky C., Giere I., Van Thillo G. (2012). Planned pregnancy in a chronic myeloid leukemia patient in molecular remission. *Case Reports in Hematology*.

[2] Alizadeh H., Jaafar H., Kajtár B. (2015). Outcome of 3 pregnancies in a patient with chronic myeloid leukemia who received 3 types of tyrosine kinase inhibitors each in different pregnancy: follow-up of the case with a review of published reports. *Annals of Saudi Medicine*.

[3] Martin J., Ramesh A., Devadasan L., Palaniappan, Martin J. J. (2011). An uneventful pregnancy and delivery, in a case with chronic myeloid leukemia on imatinib. *Indian Journal of Medical and Paediatric Oncology*.

[4] Mahon F.-X., Réa D., Guilhot J. (2010). Discontinuation of imatinib in patients with chronic myeloid leukaemia who have maintained complete molecular remission for at least 2 years: the prospective, multicentre Stop Imatinib (STIM) trial. *The Lancet Oncology*.

[5] Melo J. V., Ross D. M. (2011). Minimal residual disease and discontinuation of therapy in chronic myeloid leukemia: can we aim at a cure?. *Hematology*.

[6] Abruzzese E., Trawinska M. M., De Fabritiis P., Perrotti A. P. (2014). TYrosine kinase inhibitors and pregnancy. *Mediterranean Journal of Hematology and Infectious Diseases*.

